# Diagnosis and follow-up of the first case of Gaucher disease under enzyme replacement therapy in Senegal

**DOI:** 10.4314/gmj.v60i1.7

**Published:** 2026-03

**Authors:** Mohamed Keita, Blaise F Faye, Alioune B Senghor, Alioune B Diallo, Elimane S Bousso, Sokhna A Touré, Moussa Seck, Saliou Diop

**Affiliations:** Clinical Haematology Department of National Blood Transfusion Centre, Dakar, Senegal

**Keywords:** Gaucher disease, Glucocerebrosidase deficiency, Enzyme replacement therapy, Imiglucerase (Cerezyme), Treatment outcomes

## Abstract

Gaucher disease is a rare inherited disorder caused by a deficiency of the lysosomal enzyme β-glucocerebrosidase, leading to the accumulation of glucocerebroside within macrophages. This accumulation results in a wide spectrum of clinical manifestations, which may be life-threatening in severe cases. We report the first documented case in Senegal of a patient with Gaucher disease successfully treated with enzyme replacement therapy. The diagnosis was established based on characteristic bone marrow findings showing Gaucher cells, together with markedly reduced β-glucocerebrosidase activity and elevated chitotriosidase levels. The disease was diagnosed at the age of 11 years, and regular intravenous enzyme replacement therapy was initiated. Treatment was well tolerated and resulted in significant clinical improvement and a marked enhancement in the patient's quality of life. However, genetic confirmation by GBA1 mutation analysis could not be performed due to limited access to molecular testing, which represents an important limitation of this report and prevented precise molecular classification of the disease. This case highlights the importance of early diagnosis and timely initiation of enzyme replacement therapy to prevent severe complications in Gaucher disease. Long-term follow-up studies remain necessary to further evaluate the efficacy and safety of enzyme replacement therapy in this setting.

**Funding:**

None declared

## Introduction

Gaucher disease (GD) is a rare autosomal recessive genetic disorder caused by mutations in the GBA1 gene, located on chromosome 1 (1q21). It is characterised by a deficiency of the lysosomal enzyme glucocerebrosidase, leading to the accumulation of its substrate, glucosylceramide, in macrophages.^1^ The phenotype is variable, ranging from asymptomatic to extremely serious forms that can be life-threatening in the absence of specific treatment.^2^ In countries with limited resources, such as Senegal, diagnosis is difficult due to the unavailability of assays for the enzymes involved and exploration of the GBA1 gene. Treatment with enzyme replacement therapy is inaccessible because of the high cost. This leads to an unfavourable outcome for patients. We describe the diagnostic and evolutionary parameters under treatment of the first case of Gaucher disease to benefit from enzyme replacement therapy in Senegal.

## Case Report

MF had no perinatal problems. Breastfeeding, weaning and feeding were well managed. His vaccination was complete according to the national expanded childhood immunisation program, and his psychomotor development was correct. He is the eldest of 3 siblings, the second of whom died at the age of 6 from complications of a chronic pathology characterised by splenomegaly that appeared at the age of 2, associated with chronic anaemia. His parents were not consanguineous.

MF was referred to us by his paediatrician at the age of 11 for investigation of splenomegaly. The onset was at age 2, marked by the appearance of a painless lump on the left flank that slowly increased in size. At the age of 4, the parents noticed a delay in height and weight. At the age of 8, he gradually developed physical asthenia and shortness of breath on exertion, which prevented him from attending school. This led to a consultation at Dakar University Hospital. The paediatrician diagnosed large splenomegaly and severe anaemia, which required blood transfusions every 4 months during the 3 years he spent in paediatric care.

The initial general examination revealed delayed growth and weight, with a weight of 21 kg (below the 3rd percentile) and a height of 118 cm (below the 3^rd^ percentile). The mucous membranes were pale, without icterus. Cardiovascular examination revealed regular tachycardia at 106 beats per minute associated with a diffuse systolic murmur. The abdomen was distended by painless splenomegaly extending 17 cm below the costal crest on the left mid-clavicular line, and painless hepatomegaly with a firm lower border extending 7 cm below the costal crest on the right mid-clavicular line. There were no clinically detectable neurological manifestations.

**Figure 1:**
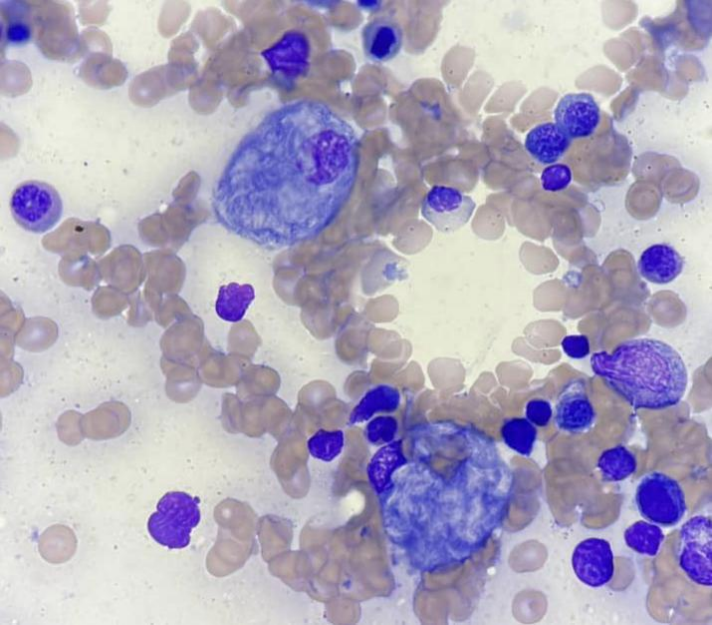
Gaucher cells (red arrow) under the light microscope (x100) after May-Grünwald Giemsa staining

The full blood count revealed anaemia with a haemoglobin level of 8.4 g/dL, associated with microcytosis (mean corpuscular volume 70 fL) and thrombocytopenia (112 Å∼10^9^/L). The white blood cell count was within the normal range (5.6 Å∼10^9^/L). The bone marrow aspiration showed large, non-cohesive cells with sharp outlines, abundant cytoplasm, moderately basophilic, fibrillar, giving the impression of crumpled paper, and an eccentric nucleus with loose chromatin. This appearance was compatible with Gaucher-type cells (Figure 1).

**Figure 2:**
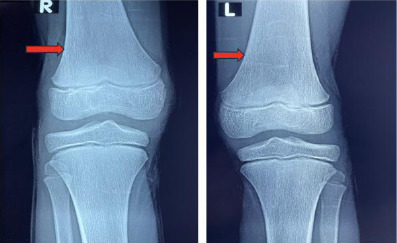
Frontal knee X-ray (R: Right; L: Left) “Erlenmeyer funnel” appearance of the lower extremities of the femurs (red arrow)

Enzyme assays revealed a beta-glucocerebrosidase deficiency of 10 pmol/min/mg [norm 20-80 pmol/min/mg protein], and an increase in chitotriosidase to 27 nmol/min/ml [normal value is less than 1.5 nmol/min/ml]. Serum ferritin was elevated to 858 ng/ml. Liver and kidney function tests were normal. Abdominal ultrasound showed homogeneous splenomegaly with a splenic arrow at 210mm and homogeneous hepatomegaly with a hepatic arrow at 130 mm. The portal trunk was of normal calibre for his age. X-rays of the knees revealed bone demineralisation associated with an “Erlenmeyer funnel” appearance of the lower extremities of the femurs (Figure 2). These findings were consistent with a diagnosis of Gaucher disease. Genetic typing was not performed.

### Therapeutic interventions

Thanks to the International Gaucher Alliance, he received enzyme replacement therapy with Imiglucerase (Cerezyme) at a dose of 60mg/kg every 15 days from 7 May 2021.

### Clinical results

**Figure 3:**
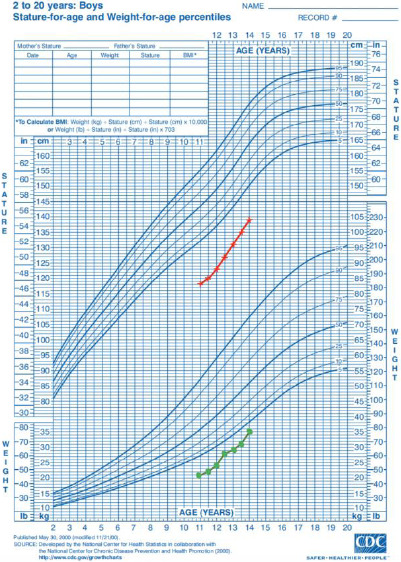
Evolution of weight (green curve) and height (red curve) under enzyme therapy according to the WHO growth standards for Senegal

**Figure 4:**
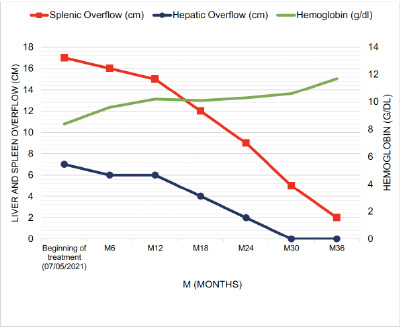
Changes in spleen and liver size and haemoglobin levels under enzyme therapy

A straightening of the stature and weight curves with a gain of 20 cm in height and 14 kg in weight after 36 months of treatment (figure 3). A significant reduction in the size of the spleen and liver. The splenic overhang was reduced from 17 cm to 2 cm on the left mid-clavicular line after thirty-six months of treatment. There was also complete regression of hepatomegaly after thirty months of enzyme therapy (Figure 4).

### Biological results

Normalisation of platelet and ferritin levels after 6 months of treatment. Progressive increase in haemoglobin to 11.7 g/dl after thirty-six months of treatment (Figure 4).

### Bone

An improvement in bone transparency and a reduction in the funnel-shaped appearance after thirty-six months of treatment.

### Patient Perspective

The parents were satisfied with the healthcare provided and hopeful about their health outcomes. No adverse or unexpected effects were observed during follow-up.

Socio-scholastic: correct completion of the school programme and active participation in the non-sporting activities of his classmates. He was in the 3^rd^ year of primary school at the time of the thirty-six-month assessment of substitution therapy.

Ethical consent was duly obtained from the internal ethical committee of the Military Hospital of Ouakam, with Registration number 024/2024/CER/HMO.

## Discussion

Diagnostic difficulties and the lack of treatment for symptomatic forms of Gaucher disease make their prognosis poor in countries with limited resources. The diagnosis is made when the patient presents a persistent splenomegaly, sometimes associated with hepatomegaly, anaemia, neurological signs, skeletal deformities and characteristic overload cells on cytological examination.^3^ It's confirmed by an enzyme assay showing a drop in β-glucosidase activity in total white blood cells, mononuclear cells, fibroblasts, and dried blood spots, combined with an increase in plasma chitotriosidase. Genetic study reveals a mutation in the GBA1 gene located on chromosome 1 (1q21). In addition to confirming the diagnosis, this mutation specifies the genotype.^4^

Three main phenotypic presentations have been identified: type 1 is the most common, accounting for 94% of all GD cases recorded by the Gaucher register. It leads to a progressive course of the disease, with the spleen, liver, and bone marrow frequently affected and, in severe cases, the lungs and kidneys. Hepatosplenomegaly and haematological complications, including anaemia and thrombocytopenia with bleeding, are common in untreated type 1 Gaucher disease (GD), which accounts for approximately 90–95% of all cases.

Acute neuropathic GD (type 2) manifests in early childhood, with rapidly progressive neurological deterioration and death usually occurring before the age of 2, representing about 1–5% of cases. Type 3 GD is characterised by slower neurological deterioration, typically presenting in childhood or adolescence and associated with the systemic manifestations of type 1; it accounts for approximately 5% of Gaucher disease cases.^5^ In our patient, it was not possible to perform genetic testing to determine the genotype. However, his very progressive symptoms, with no neurological manifestations, led us to classify him as type I. The first-line treatment for symptomatic type 1 and type 3 Gaucher disease is enzyme replacement therapy (imiglucerase), whereas none of the enzyme replacement therapies is indicated for type 2 Gaucher disease, since treatment has no impact on the rapid progression of its severe neurological symptoms.^6^ In type 1 GD, enzyme replacement therapy breaks down the glucosylceramide accumulated in the tissues, thereby improving haematological abnormalities, hepatosplenomegaly and patients' quality of life.^7^

Six months after the start of enzyme replacement therapy with Imiglucerase (Cerezyme) at a dose of 60 mg/kg every 15 days, the size of the patient's liver and spleen began to decrease; haemoglobin levels improved progressively, and ferritin levels returned to normal. Similarly, studies have shown an improvement in haematological and visceral parameters over 6 months with a similar dosage.^8^

After one year of enzyme therapy, the size of the patient's liver and spleen had decreased considerably and had almost returned to normal after 2 years of treatment; haemoglobin levels improved progressively but remained below normal levels. In addition, a clear improvement in statural and weight parameters was observed after 36 months of enzyme replacement therapy; however, the patient's height and weight remained below the 5th percentile according to WHO growth standards. This can be explained by the fact that type 1 GD requires long-term treatment (on average 20 years) with imiglucerase to maintain sustained efficacy in correcting haematological, visceral, and certain bone symptoms.^9^ Our patient benefited from 36 months of substitutive treatment, resulting in a favourable outcome and a return to school activities. Continued enzyme therapy could therefore enable him to further improve his clinical condition. Fifteen per cent of patients treated with imiglucerase develop anti-imiglucerase antibodies during the first 6 months of treatment; hypersensitivity has also been reported in 3% of patients.^10^ Our patient showed no signs of therapeutic ineffectiveness.

A major limitation of this case report is the absence of genetic confirmation by GBA1 mutation analysis, which was not available in our setting. Although enzymatic and clinical findings were sufficient to establish the diagnosis, genetic testing would have allowed precise genotypic characterisation and further prognostic assessment.

## Conclusion

Gaucher disease is a rare lysosomal storage disorder that remains underdiagnosed in many developing countries and is associated with a poor prognosis in the absence of enzyme replacement therapy. This case report demonstrates the efficacy and safety of enzyme replacement therapy in the first reported patient with Gaucher Disease treated in Senegal. Improved availability of diagnostic tools and access to enzyme replacement therapy could substantially enhance patient outcomes in resource-limited settings.
